# Non-operative management of acute appendicitis is not readily generalisable to Honduras: a viewpoint from a low-resource setting

**DOI:** 10.7189/jogh.16.03019

**Published:** 2026-07-24

**Authors:** Marlon Rodriguez Valladares, Paola Zuniga, Marlen Flores, Valeria Loja Pauta, Cosman Camilo Mandujano

**Affiliations:** 1 Facultad de Ciencias Médicas, Universidad Nacional Autónoma de Honduras, Tegucigalpa, Francisco Morazán, Honduras; 2 Facultad de Medicina y Cirugía, Universidad Católica de Honduras ‘Nuestra Señora Reina de la Paz’, San Pedro Sula, Cortés, Honduras; 3 Facultad de Ciencias Médicas, Universidad de Cuenca, Cuenca, Azuay, Ecuador; 4 Department of Surgery, Montefiore Medical Center, Bronx, New York, USA

**Keywords:** appendicitis, global surgery, health system strengthening, Honduras, low- and middle-income countries, non-operative management

## Abstract

Non-operative management (NOM) of acute appendicitis has been validated by trials such as Appendicitis Acuta and Comparison of Outcomes of Antibiotic Drugs and Appendectomy, with treatment failure rates of approximately 25–30% within one year, raising hopes that a less invasive approach could become the standard of care. However, the conditions underpinning these trials, such as early presentation, reliable imaging, uninterrupted access to antibiotics, structured follow-up, and immediate surgical backup, are not consistently available in Honduras. Up to 54% of patients at a Honduran tertiary centre present with gangrenous or perforated disease, considerably reducing the proportion of patients eligible for NOM, while sociocultural practices such as abdominal manipulation (*sobada*), weak referral networks, and limited diagnostic capacity further delay access to care. We argue that NOM cannot currently be recommended as a standard approach across the Honduran health system, although it may have a role in well-resourced tertiary centres. Importantly, this is not a rejection of NOM evidence: the primary drivers of poor outcomes in Honduras, late presentation and limited system capacity, are not addressed by NOM and could worsen if applied without adequate infrastructure. We propose that strengthening point-of-care diagnostics, referral pathways, antibiotic supply chains, and follow-up capacity should be the immediate priority, both to improve outcomes directly and to build the foundations for selective NOM implementation as the health system evolves.

Non-operative management (NOM) of acute appendicitis has gained widespread attention as an alternative to appendectomy following randomised clinical trials demonstrating safety in selected patients [1-4]. However, the generalisability of these findings to low- and middle-income countries (LMICs) remains uncertain, as the conditions required for safe NOM implementation are often absent in these settings [5,6]. Honduras represents a high-burden context in which structural, sociocultural, and health system factors differ considerably from those of the settings where NOM has been validated. Global Burden of Disease data indicate that Honduras has age-standardised appendicitis disability-adjusted life years rates substantially above the Latin American regional average, with a disproportionate burden concentrated in individuals under 50 years of age compared with high-income countries [7,8]. Although uncertainty remains regarding the precision of country-level estimates, these data suggest that Honduras represents a relevant setting for examining the transferability of NOM evidence.

We argue that NOM, while effective in controlled settings, cannot currently be recommended as a standard approach across Honduras. This position is not a rejection of NOM evidence, nor does it dismiss the potential role of NOM in specific, well-equipped tertiary centres that may already possess some of the required infrastructure. Rather, it is a recognition that the primary drivers of poor outcomes in Honduras, late presentation and limited health system capacity, are not addressed by NOM protocols and may even be exacerbated by them when applied without the necessary supporting conditions. Crucially, NOM and health system strengthening are not competing priorities; as diagnostics, referral pathways, follow-up capacity, and surgical infrastructure improve, the feasibility and safety of NOM in Honduran settings may likewise increase.

## EVIDENCE BASE AND KEY ASSUMPTIONS

Landmark trials such as Appendicitis Acuta and Comparison of Outcomes of Antibiotic Drugs and Appendectomy demonstrated that antibiotics can safely treat uncomplicated appendicitis in carefully selected patients, with treatment failure rates of approximately 25–30% within one year [1–4]. Importantly, these trials primarily establish efficacy under protocol-controlled conditions rather than effectiveness under routine clinical practice. Therefore, the main question in Honduras is not whether NOM can work under ideal circumstances, but whether the conditions required for its safe and effective implementation are consistently present across the healthcare system. A potential NOM trial in Honduras would rely on several key assumptions, such as early presentation, reliable imaging to confirm uncomplicated disease, uninterrupted antibiotic supply, structured follow-up, and immediate surgical backup in case of failure (**Table 1**).

**Table 1:** Mapping of key NOM trial assumptions against available evidence from the Honduran healthcare context

NOM trial assumption	Honduran healthcare reality
Early presentation within 48–72 hours	Substantial proportion present with advanced disease; population-level presentation timing data unavailable
Reliable imaging (CT or ultrasound) to confirm uncomplicated disease	CT access severely limited; ultrasound availability inconsistent outside tertiary centres; radiological interpretation capacity variable
High-quality, uninterrupted antibiotic supply	Supply chain disruptions documented; antibiotic quality and availability inconsistent, particularly in peripheral facilities
Structured follow-up with clear re-presentation criteria	Weak referral networks; geographic, financial, and transportation barriers impede follow-up; no formal NOM follow-up protocols exist
Immediate surgical backup available for NOM failures	Surgical capacity concentrated in tertiary centres; emergent re-operation for NOM failures may not be rapidly accessible in rural settings
Patient ability to recognise and report deterioration	Health literacy gaps; cultural practices may mask or delay recognition of clinical deterioration

CT – computed tomography, NOM – non-operative management

Moreover, NOM has been proposed as a potentially valuable option even in resource-constrained settings, precisely because it may reduce the number of surgical procedures, operating theatre time, and hospital stay in patients with uncomplicated disease [5]. In the African context, a review highlighted that antibiotic-first treatment could preserve both human and material resources in LMICs with limited surgical capacity [5]. Similarly, where early, imaging-confirmed uncomplicated appendicitis is identifiable and short inpatient antibiotic courses are feasible, some settings with functioning referral networks and adequate antibiotic supply could derive benefit from selective NOM in carefully chosen patients. These considerations reinforce the importance of a stratified implementation rather than blanket adoption or blanket rejection: the question is not whether NOM has any role in resource-limited contexts, but under which specific conditions that role can be exercised safely.

## CONTEXTUAL MISMATCH IN HONDURAS

**Figure 1:**
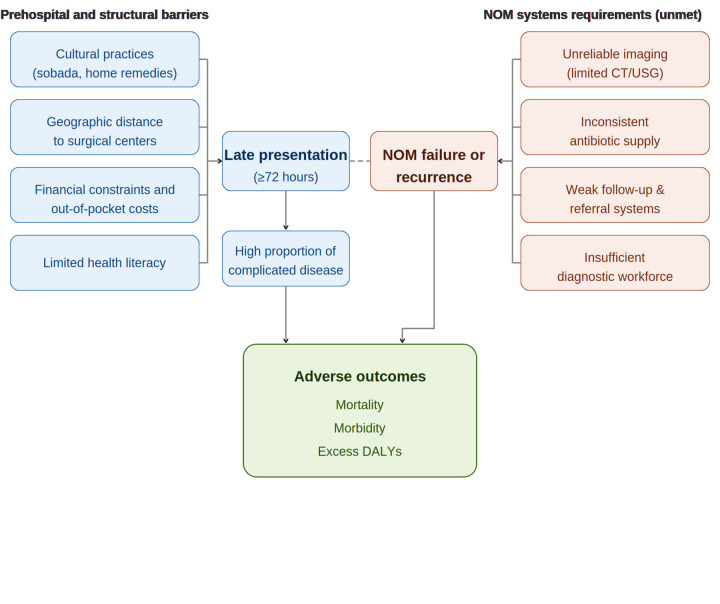
Conceptual causal pathway: barriers to safe non-operative management implementation in Honduras. CT – computed tomography, DALYs – disability-adjusted life years, NOM – non-operative management, USG - ultrasound.

Many patients in Honduras present with more advanced forms of appendicitis, including gangrenous or perforated disease. A hospital-based study at a tertiary centre documented a high burden of complicated appendicitis at admission, with 54% of cases presenting as gangrenous or perforated disease [9]. Given the referral nature of the institution, this proportion may be higher than observed in the broader Honduran population. Because NOM is indicated only for uncomplicated cases, widespread late presentation sharply limits the pool of eligible patients and reduces the strategy’s population-level impact. It should be acknowledged that the relative contributions of delayed presentation, diagnostic limitations, surgical capacity constraints, perioperative care availability, and socioeconomic factors to poor outcomes remain incompletely quantified at the population level in Honduras. Population-based data on staging at presentation remain scarce, representing a critical evidence gap. These interrelated barriers could influence feasibility, safety, and potential impact of NOM implementation in Honduras (**Figure 1**).

Sociocultural and structural factors further compound delays. In some communities, particularly in rural and peri-urban areas, abdominal pain is initially managed through traditional practices, such as abdominal manipulation (*sobada*) by family members or healers. This prehospital pathway has been associated with complicated appendicitis in paediatric patients and not accounted for in NOM trials, which are based on settings with prompt access to formal care [10]. The national prevalence of sobada, its geographic distribution, and its relative contribution to presentation delay compared with other barriers, transportation difficulties, financial constraints, and limited healthcare access, have not been systematically quantified. Available evidence linking the practice to complicated appendicitis derives from a single paediatric study in Honduras [10]; it should therefore be interpreted as hypothesis-generating rather than conclusive. These cultural, geographic, and financial barriers likely interact, and their relative weight across different regions of the country warrants prospective investigation.

Health system limitations add another layer of risk. Safe NOM demands reliable diagnostic imaging, uninterrupted antibiotic supply, and rapid access to surgery upon failure – all of which are inconsistent, particularly outside major urban centres [6,11]. Furthermore, surgical management also depends on substantial infrastructure, including functioning operating theatres, anaesthesia services, trained surgeons, blood product availability, and postoperative care capacity. Both approaches carry resource requirements that are unevenly distributed across Honduras. The comparative advantage of early appendectomy in the current Honduran context lies in its ability to provide definitive source control at a single encounter, without relying on follow-up or repeat imaging. In contrast, NOM leaves the appendix in place, exposing patients to the possibility of recurrence or progression to advanced disease when follow-up is unreliable [12].

Honduras is heterogeneous: tertiary centres in Tegucigalpa and San Pedro Sula have better infrastructure, while rural and peri-urban facilities face severe constraints. Outcomes and feasibility of NOM also differ by age group, with less established evidence in children [1–4]. Any future application of NOM would therefore require explicit stratification by facility level, geographic region, and patient population rather than uniform national adoption. Well-resourced tertiary centres in major urban areas may already possess several prerequisites for piloting NOM in carefully selected patients, whereas peripheral and rural facilities currently lack the diagnostic and follow-up infrastructure to do so safely. The distinction between what is feasible at a given centre and what is generalisable across the national health system is the core of the policy question addressed here.

## IMPLICATIONS FOR PRACTICE AND POLICY

Applying NOM protocols without the necessary infrastructure and follow-up systems could carry unintended risks, including delayed recognition of treatment failure and overestimation of system readiness for safe implementation. In such circumstances, emphasis on NOM could inadvertently divert attention from broader structural barriers that continue to drive poor outcomes. The evidence from high-income trials should guide patient selection only within systems capable of safely managing failures, not as a universal call to reduce surgical rates. A critical but underexplored challenge is diagnostic uncertainty, as reliable discrimination between uncomplicated and complicated appendicitis is a prerequisite for NOM eligibility. However, this distinction depends on imaging modalities that are inconsistently available across Honduras. The principal limitation likely involves a combination of restricted access to ultrasound and computed tomography, variable interpretation capacity, and an insufficiently trained radiology workforce, particularly in non-urban settings. Any realistic NOM pathway must explicitly account for these diagnostic constraints rather than assuming that clinical and laboratory criteria alone can reliably exclude complicated disease.

## PRIORITIES FOR IMPROVING OUTCOMES

The central challenge in Honduras is not overtreatment but delayed care. Future efforts should prioritise system-level interventions that enable earlier intervention:

1. Community education on warning signs of acute abdominal illness, coupled with engagement of traditional healers and community health workers to shorten prehospital delays.
2. Strengthened referral networks to ensure prompt transfer from peripheral facilities to surgical centres.
3. Expansion of diagnostic capacity through point-of-care ultrasound (POCUS), which has demonstrated feasibility and acceptable accuracy as a first-line tool in LMIC settings when performed by trained providers [13]. Realising the potential of POCUS will, however, require sustained investment in workforce training, competency-based credentialing, equipment maintenance, and quality assurance mechanisms to ensure diagnostic accuracy under routine clinical conditions rather than only in supervised research settings.

Future research should focus on quantifying presentation delays, identifying modifiable barriers, and evaluating context-specific interventions. A priority is generating reliable population-level epidemiological data on the proportion of patients presenting with uncomplicated *vs* complicated appendicitis across different facility levels and regions; without this, it is impossible to estimate the true size of the potential NOM-eligible population or the strategy’s achievable impact. If NOM is to be studied locally, we propose a prospective multicentre observational cohort study enrolling patients across tertiary, secondary, and peripheral facilities, stratified by region and facility level. Such a study should measure real-world eligibility rates, treatment failure rates, follow-up adherence, and clinical outcomes, providing the context-specific evidence base required before any broader implementation could be responsibly considered.

## CONCLUSIONS

Our findings do not challenge NOM as a clinical strategy but caution against its premature generalisation to the Honduran healthcare system, which lacks the conditions required for its safe delivery. Strengthening point-of-care diagnostics, referral networks, antibiotic supply chains, and follow-up capacity addresses the root causes of poor appendicitis outcomes in Honduras while simultaneously building the infrastructure that would make NOM viable in the future. The immediate priority is earlier, more reliable surgical care for all patients; a selective, evidence-based role for NOM can follow as those foundations are established.

## Data Availability

**Data availability:** No data were generated during the writing of this viewpoint.
